# Paternal cocaine-seeking motivation defines offspring’s vulnerability to addiction by down-regulating GABAergic GABRG3 in the ventral tegmental area

**DOI:** 10.1038/s41398-024-02835-w

**Published:** 2024-02-22

**Authors:** Jian Cui, Nan Huang, Guangyuan Fan, Tao Pan, Kunxiu Han, Changyou Jiang, Xing Liu, Feifei Wang, Lan Ma, Qiumin Le

**Affiliations:** 1 School of Basic Medical Sciences, State Key Laboratory of Medical Neurobiology, MOE Frontiers Center for Brain Science, Institutes of Brain Science, Department of Neurology, Pharmacology Research Center, Huashan Hospital, Fudan University, Shanghai, China; 2 Research Unit of Addiction Memory, Chinese Academy of Medical Sciences (2021RU009), Shanghai, China

**Keywords:** Molecular neuroscience, Epigenetics and behaviour, Epigenetics and plasticity, Addiction

## Abstract

Epidemiological investigations indicate that parental drug abuse experiences significantly influenced the addiction vulnerability of offspring. Studies using animal models have shown that paternal cocaine use and highly motivated drug-seeking behavior are important determinants of offspring addiction susceptibility. However, the key molecules contributing to offspring addiction susceptibility are currently unclear. The motivation for cocaine-seeking behavior in offspring of male rats was compared between those whose fathers self-administered cocaine (SA) and those who were yoked with them and received non-contingent cocaine administrations (Yoke). We found that paternal experience with cocaine-seeking behavior, but not direct cocaine exposure, could lead to increased lever-pressing behavior in male F1 offspring. This effect was observed without significant changes to the dose-response relationship. The transcriptomes of ventral tegmental area (VTA) in offspring were analyzed under both naive state and after self-administration training. Specific transcriptomic changes in response to paternal cocaine-seeking experiences were found, which mainly affected biological processes such as synaptic connections and receptor signaling pathways. Through joint analysis of these candidate genes and parental drug-seeking motivation scores, we found that gamma-aminobutyric acid receptor subunit gamma-3 (*Gabrg3*) was in the hub position of the drug-seeking motivation-related module network and highly correlated with parental drug-seeking motivation scores. The downregulation of *Gabrg3* expression, caused by paternal motivational cocaine-seeking, mainly occurred in GABAergic neurons in the VTA. Furthermore, down-regulating GABAergic *Gabrg3* in VTA resulted in an increase in cocaine-seeking behavior in the Yoke F1 group. This down-regulation also reduced transcriptome differences between the Yoke and SA groups, affecting processes related to synaptic formation and neurotransmitter transmission. Taken together, we propose that paternal cocaine-seeking behavior, rather than direct drug exposure, significantly influences offspring addiction susceptibility through the downregulation of *Gabrg3* in GABAergic neurons of the VTA, highlighting the importance of understanding specific molecular pathways in the intergenerational inheritance of addiction vulnerability.

## Introduction

Drug addiction is a chronic rehabilitating disease, characterized by uncontrollable drug use and compulsive drug-seeking behavior [1]. In addition to individual differences in addiction susceptibility [2, 3] detected at gene level [4, 5], the phenomenon of familial aggregation of addiction found in epidemiologic investigations [6–9] suggests the possibility of intergenerational inheritance, and was supported by human investigations [6–11]. Research has shown a strong association between paternal drug addiction and offspring vulnerability to addiction in animal models [12–15]. Interestingly, while the effect of the mother’s experience of addiction or drug exposure was previously thought to be more significant, especially during pregnancy [16], recent studies have found evidence of paternal inheritance as well [17]. In animal models, paternal addictive drug exposure has been found to significantly affect addiction susceptibility [18, 19], mood [20], sociability [21], learning, and cognitive functions [22, 23], etc. in offspring.

Understanding the mechanism by which addictive substances affect offspring and identifying key intervention targets can play a key role in combating the adverse effects of drug addiction on future offspring. Despite the numerous findings, it is important to note that drug exposure, acting as a novel chemical, is producing different and even opposite outcomes in offspring compared to the neuropsychological effects of drug reinforcement. We previously used a rat cocaine self-administration model to demonstrate that offspring produced by paternal experiences of voluntary cocaine-seeking, rather than those receiving passive drug infusions, produce sires that exhibit higher cocaine self-administration behaviors, but the mechanism is not unveiled [19]. Therefore, it is imperative to further explore the reasons for the emergence of increased drug-seeking behavior in offspring, and meanwhile propose the potential molecular mechanisms behind it.

As addictive drug-seeking behavior develops, drug-seeking motivation gradually intensifies, leading to a series of neuroplasticity changes in the midbrain limbic reward circuit during chronic drug exposure, as well as drug withdrawal and relapse [24–28]. In an effort to disentangle drug seeking, leading to vulnerability to drug reinforcement, from “drug exposure” factor, leading to protective effects of cocaine resistance, we utilized our previous model and compared transcriptomic changes, dopamine level of offspring sired by voluntary and passive cocaine use in multiple brain regions [29]. Of the screened regions, the ventral tegmental area (VTA) exhibited changes more strongly associated with the paternal voluntary cocaine-seeking experience. The VTA is known to be primarily responsible for dopamine release and motivation regulation, playing a pivotal role in reward sensation and motivation reinforcement during the development of addictive behaviors [30–34].

Hence, to investigate how parental motivation for drug-seeking experiences affects the neural system of offspring and determines their vulnerability to drug addiction, we randomly assigned male rats to three groups, cocaine self-administration (Coc-SA), yoked administration (Coc-Yoke), and saline self-administration (Sal-SA), and used them to generate F1. Behavioral assessment and transcriptome sequencing were conducted on the ventral tegmental area, and the data were analyzed using Weighted Gene Co-Expression Network Analysis (WGCNA) to correlate behavior with transcriptome changes. Our current findings demonstrate that the expression of Gabrg3 in VTA GABAergic neurons of offspring with a history of motivational cocaine-seeking behavior is downregulated. This downregulation leads to remodeling of the transcription network in the VTA, ultimately resulting in heightened cocaine-seeking motivation in the offspring.

## Materials and methods

The detailed methods used in this study are further detailed in the supplemental information. Sprague-Dawley rats and their male offspring were used for all experiments. Self-administered rats were paired with yoked-administered rats one by one during the 30-day cocaine self-administration training. After the final self-administration training, each selected F0 rat was mated with two naive female rats to generate F1 offspring. The male F1 rats were then subjected to behavior tests and transcriptome sequencing of the VTA. Bioinformatics analysis was conducted, and the hub gene associated with motivational drug-seeking behavior in male offspring was validated. The function of the hub gene was verified by chemogenetic regulation in male offspring.

## Results

### Paternal motivation to seek cocaine is passed on to F1 male offspring

**Fig. 1:**
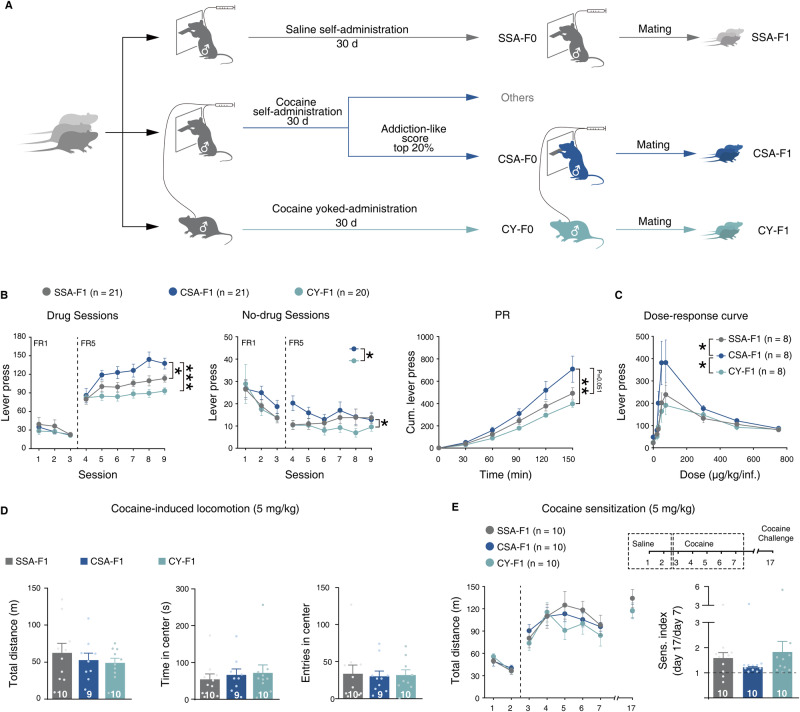
The inheritance of vulnerability to cocaine addiction depends on the motivation for cocaine self-administration. **A** Diagram illustrating the process of cocaine self-administration and mating. **B** Performance of male F1 rats in cocaine self-administration. Left: number of lever presses in drug sessions under an FR program; middle: number of lever presses in no-drug sessions under an FR program; Right: cumulative number of lever presses under a PR program. SSA-F1, *n* = 21; CSA-F1, *n* = 21; CY-F1, *n* = 20. **C** Dose-response curves for cocaine in male F1 rats. SSA-F1, *n* = 8; CSA-F1, *n* = 8; CY-F1, *n* = 8. **D** Locomotor activity of naive male F1 rats after receiving 5 mg/kg i.p. injections of cocaine. Left: total distance traveled; middle: time spent in the center area; right: number of entries to the center area. SSA-F1, *n* = 10; CSA-F1, *n* = 9; CY-F1, *n* = 10. **E** Behavioral sensitization in male F1 rats after receiving 5 mg/kg doses of cocaine. Left: total distance traveled; right: sensitization index. SSA-F1, *n* = 10; CSA-F1, *n* = 10; CY-F1, *n* = 10. Results are shown as mean ± s.e.m., ***P < 0.001, **P < 0.01, *P < 0.05.

In order to better differentiate the effects of active cocaine-seeking behavior from noncontingent cocaine exposure, three groups were used, Sal-SA, Coc-SA, and Coc-Yoke, where Coc-Yoke was paired one by one with Coc-SA rats, without access to levers or drug-paired signals, meanwhile received cocaine injections at the same time at the same dose. Self-administering rats underwent a 32-day training paradigm (5 days at a fixed ratio of 1 infusion per lever pressed (FR1), 10 days FR5, 10 days FR5, the FR5 sessions were interspersed with two progressive-ratio (PR) tests, Supplementary Fig. S1A–D). By scoring Coc-SA rats on counts of lever pressed during FR, no-drug period, and performance on the two PR tests, we found that about 20% of the top-ranked rats exhibited different, higher drug-seeking behaviors compared with other individuals (Supplementary Fig. S1E, F). Based on previous findings that only the offspring of individuals with high drug-seeking motivation demonstrated high addiction susceptibility, we selected the top 20%, their paired Coc-Yoke, and seven randomly selected litters of Sal-SA individuals as CSA-F0, CY-F0, and SSA-F0, respectively, to obtain F1 generation (Fig. 1A).

There were no significant differences in litter size or sex distribution among all groups of male F1 rats (Supplementary Fig. S2A). Moreover, their body weight at 3 and 7 weeks of age did not vary significantly (Supplementary Fig. S2B). We also observed no differences in the performance of the food training task, novel object recognition test, open field test, elevated plus maze, or Y-maze between the three groups of male F1 rats (Supplementary Fig. S2C–K).

Despite similar performances in locomotion, anxiety-like behavior, and memory tasks across the three groups, the male CSA-F1 exhibited higher lever presses during the drug sessions of the FR5 program, compared to SSA-F1 and CY-F1 (Fig. 1B, left). CSA-F1 rats also exhibited higher compulsivity and cocaine-seeking motivation, as indicated by lever presses during the no-drug sessions of the FR5 program (Fig. 1B, middle) and cumulative lever presses in the PR program (Fig. 1B, right), compared to CY-F1 rats.

Higher sensitivity to cocaine could also influence drug-seeking behavior. In dose-response tests, CSA-F1 rats displayed higher lever presses than SSA-F1 and CY-F1 rats at descending doses (750, 500, 300, 75, 50, 30, 22.5, 0 μg kg inf^−1^), but their sensitivity to cocaine did not change, indicated by the maximum responsive dose of each group (Fig. 1C). Nonetheless, we did not observe any differences in cocaine-induced locomotor activity in the three groups of male F1 rats in either low dose (5 mg/kg) or high dose (10 mg/kg) (Fig. 1D and Supplementary Fig. S3A), or cocaine sensitization index in either dose (Fig. 1E and Supplementary Fig. S3B). Finally, the cocaine-induced locomotor activity test was performed after cocaine self-administration training at either a low dose (5 mg/kg) or high dose (10 mg/kg) (Supplementary Fig. S3C). Except for the decrease in time spent exploring the central area between SSA-F1 and CY-F1 rats, we did not observe any behavioral differences between the three groups of male F1 rats. Taken together, we conclude that the main factor causing increased lever pressing in CSA-F1 rats is not a change in sensitivity to cocaine, but rather an increased drug-seeking motivation.

### WGCNA analysis of transcriptome in VTA of male F1 rats reveals gene modules correlated with paternal motivational drug-seeking experiences

**Fig. 2:**
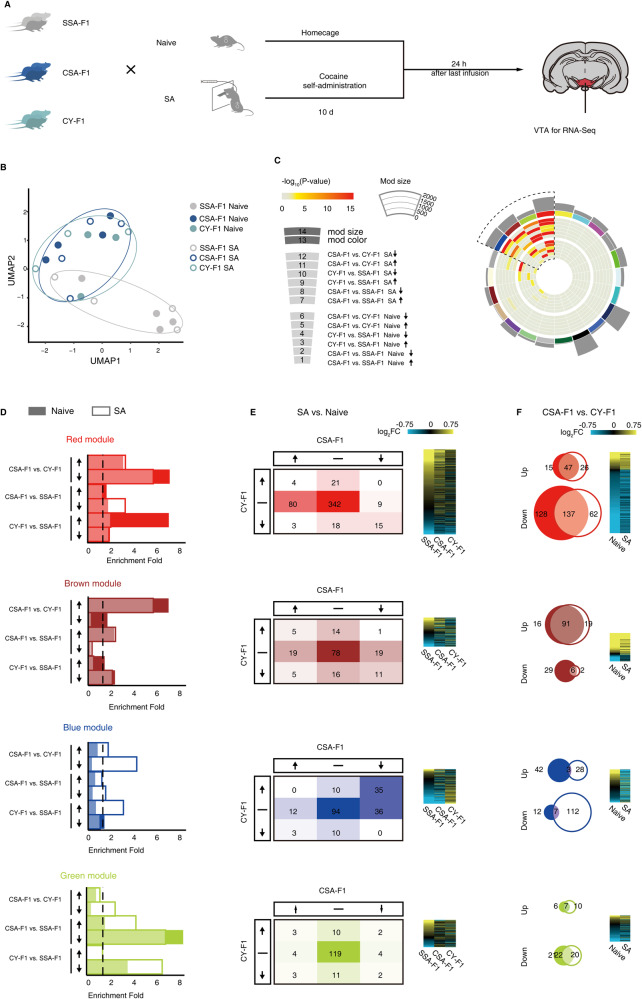
WGCNA analysis of VTA transcriptome reveals distinct behavioral traits related to gene modules. **A** Schematic of experimental design. Rats were divided into six groups (*n* = 4/group) based on the drug-seeking experience of the F0 and F1 rats. Rats from each group of SSA-F1, CSA-F1, and CY-F1 were randomly assigned to perform cocaine self-administration (Coc. SA) or remained in home cage (Naive). Twenty-four hours after the last SA training, microdissections of seven reward-associated brain regions in three groups of F1 generation under Naive and Coc. SA states were collected and subjected to mRNA-seq. **B** Clustering of F1 individuals by UMAP based on Variance Stabilizing Transformed (VST) gene expression. Colors represent groups (SSA-F1, CSA-F1, CY-F1), fills represent treatment (Coc. SA, Naive). **C** Circos plots for the WGCNA result. Each slice in the chart represents a gene co-expression module, with the outermost representing the modular size of the module. The secondary outer rectangle indicates an arbitrary color used to name the module. The inner concentric circles represent the degree of enrichment for pattern genes, with colors reflecting Fisher’s exact test (FET) *P* values. Dashed lines, modules predicted to be affected by paternal cocaine use. **D** DEG enrichment analysis of the four modules. Enrichment = (Count _(DEGs ∩ module)_/Count _module_)/(Count _module_/Count _background_). **E** Data table and union heatmap showing gene expression changes of the four modules in the naive and SA state. **F** Heatmap and Venn diagram showing expression differences between CSA-F1 and CY-F1 rats in the naive and SA state.

We performed transcriptome sequencing in the VTA of SSA-F1, CSA-F1, and CY-F1 in the naive state (in their home cages) as well as in the SA state (24 h after 10 days of cocaine self-administration) to better understand the inherited traits and adaptive changes in response to SA in CSA-F1 (Fig. 2A). Dimensionality reduction analysis of the transcriptome using UMAP (Uniform Manifold Approximation and Projection for Dimension Reduction) revealed that paternal exposure to cocaine (SSA-F1 vs. CSA-F1 and CY-F1), rather than cocaine self-administration training in F1 rats itself (naive vs. SA), produced more pronounced changes in the VTA transcriptome of F1 offspring (Fig. 2B).

Pairwise comparisons of differentially expressed genes (DEGs) across states (Supplementary Table S1), and DEGs between groups of offspring rats in the naive state and after self-administration training were used for further analysis (Supplementary Fig. S4B, C). Under naive state (Supplementary Fig. S4B, C, upper), the number of DEGs between rats in the CSA-F1 and CY-F1 groups was significantly higher than that of CY-F1 vs. SSA-F1 or CY-F1 vs. SSA-F1 (CSA-F1 vs. CY-F1, 132 up and 285 down; CSA-F1 vs. SSA-F1, 161 up and 99 down; CY-F1 vs. SSA-F1, 51 up and 81 down). After self-administration training (Supplementary Fig. S4B, C, lower), the number of DEGs was significantly increased between all groups of offspring rats compared to the naive state (CSA-F1 vs. CY-F1, 259 up, 438 down; CSA-F1 vs. SSA-F1, 222 up, 337 down; CY-F1 vs. SSA-F1, 197 up, 149 down). As summarized in Supplementary Fig. S4C, although under UMAP analysis, CSA-F1 and CY-F1 were overlapping, significant enrichment of DEGs between CSA-F1 and CY-F1 exists in VTA. Furthermore, these differences are innate and amplified after undergoing cocaine self-administration training.

To extract meaningful expression patterns from the comparisons described above, we used WGCNA [35] to construct co-expression modules. We generated 22 discrete modules and assigned an arbitrary color to name each (Fig. 2C). Four modules (red, brown, blue, and green) stood out because they contained the most DEGs (Fig. 2C and Supplementary Fig. S4D). We then performed modular expression profiling to observe potential differences among these four modules (Fig. 2D–F).

**Fig. 3:**
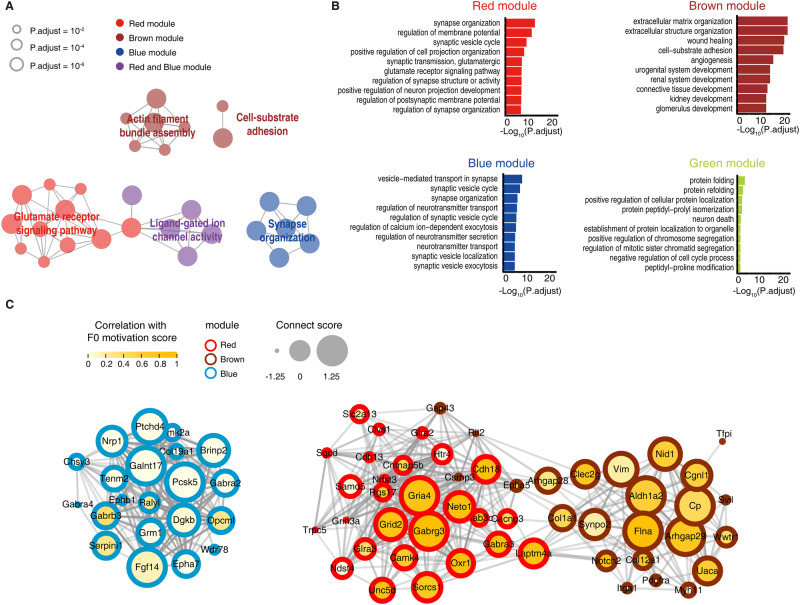
Screening of hub genes that regulate drug-seeking motivation in male F1 rats. **A** Pathway clustering of genes in four modules. The size of the circle reflecting FET *P* values, the colors represent the types of genes contained in the GO term. **B** Top 10 enriched GO terms of four modules. **C** Gene co-expression network of DEGs was plotted. The border of the circle represents the gene’s module. The color of the nodes represents the correlation between gene expression in male F1 rats and the F0 motivation score. The size of nodes represents the connect score of genes in the network.

In the red module, we observed a significant enrichment of downregulated DEGs in CSA-F1 rats compared to CY-F1 rats in both naive and SA states (Fig. 2D, first lane). Specifically, 44.3% of DEGs showed consistent direction of changes across naive and SA states (184/415, 44.3%, Fig. 2F, first lane), and 78.8% of DEGs showed reduced expression in CSA-F1 rats compared to CY-F1 rats (327/415, 78.8%, Fig. 2F, first lane). On the other hand, the brown module showed enrichment of upregulated DEGs in CSA-F1 rats compared to CY-F1 rats in both naive and SA states (Fig. 2D, second lane). In this module, 59.5% of DEGs showed consistent direction of changes in across naive and SA states (97/163, Fig. 2F, second lane), and 77.3% of DEGs showed increased expression in CSA-F1 rats compared to CY-F1 rats (126/163, Fig. 2F, second lane). In the blue module, a significant enrichment of downregulated DEGs was observed in CSA-F1 rats compared to CY-F1 rats, but most of these DEGs appeared after SA (Fig. 2D, third lane). Specifically, 64.2% of DEGs showed reduced expression in CSA-F1 rats compared to CY-F1 rats (131/204, 64.2%, Fig. 2E, third lane), and only 4.9% of DEGs showed consistent changes in expression direction across naive and SA states (10/204, 4.9%, Fig. 2F, third lane). The green module showed enrichment of DEGs in either CSA-F1 or CY-F1 rats compared to SSA-F1 rats (Fig. 2D–F, fourth lane). Moreover, GO (Gene Ontology) analysis reveals different enriched terms within each module (Fig. 3A, B). While both the red and blue modules were associated with synapse organization, and they share functional control in ligand-gated channel activity, the brown module was self-clustered and implied changes in the extracellular matrix, while the terms associated with the green module were not significantly clustered (Fig. 3B).

To summarize, the green module presented transcriptomic changes associated with parental drug exposure, while the other modules were associated with parental drug-seeking experience, which is our focus. The red and brown modules show stable transcriptome changes, while the red and blue modules show down-regulation. Additionally, different genetic modules carry very different functions. Among four modules, changes in the red module best summarizes the overall stable and down-regulated transcriptional change profile of the VTA (Supplementary Fig. S4B, C).

### Significant down-regulation of *Gabrg3* of VTA underlies increased incentive motivation for cocaine in CSA-F1

Tracing back the source of genes contributing to the enrichment term “membrane potential” (Fig. 3A, B), as enriched from the red module, revealed significant enrichment of DEGs belonging to the subunits of the glutamate receptor complex and GABA receptor families (Supplementary Fig. S6). To identify the most critical and representative genes for further biological validation, we constructed the weighted gene expression network, based on the connectivity of each gene in the network, and correlated it with the paternal motivation scores to assess potential effects of paternal drug-seeking motivation on offspring (Fig. 3C). *Gabrg3* and *Gria4*, scoring high on both scales, were chosen as alternative genes for subsequent validation.

**Fig. 4:**
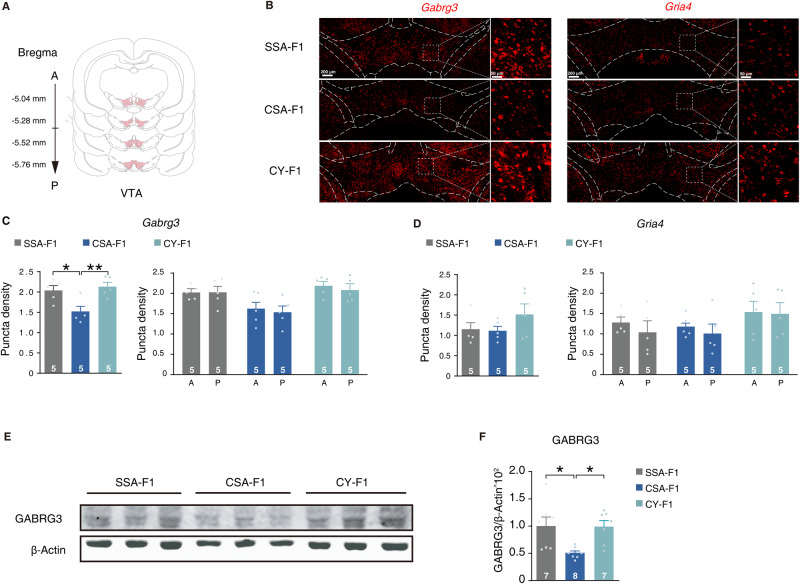
Down-regulation of *Gabrg3* in the VTA of CSA-F1 rats. **A** Schematic of sites imaged for quantification across VTA. **B** Representative smFISH images of *Gabrg3* and *Gria4* in the VTA of naive F1 offspring. Scale bar, 200 μm. **C**, **D** Quantification of total and anterior-posterior *Gabrg3* (**C**) and *Gria4* (**D**) mRNA levels in VTA of CSA-F1, CY-F1, and SSA-F1 rats using smFISH. SSA-F1, *n* = 5; CSA-F1, *n* = 5; CY-F1, *n* = 5. **E**, **F** Representative western blots (**E**) and quantification (**F**) of GABRG3 protein levels in VTA of CSA-F1, CY-F1, and SSA-F1 rats. SSA-F1, *n* = 7; CSA-F1, *n* = 8; CY-F1, *n* = 7. Results are shown as mean ± s.e.m., ***P < 0.001, **P < 0.01, *P < 0.05.

Previous studies have shown that the VTA is a heterogeneous area, where the anterior and posterior regions may contribute differently to drug-seeking behavior [36–39]. To verify the transcriptional differences between the CSA-F1 and the CY-F1 rats in vivo, we conducted single-molecule RNA fluorescence in situ hybridization (smFISH) of *Gabrg3* or *Gria4* in male F1 rats (Fig. 4A–D). We observed no changes in the expression level of *Gabrg3* or *Gria4* between the anterior and posterior VTA in each group (Fig. 4C, D, right). However, the expression level of *Gabrg3* in the whole VTA of the CSA-F1 rats was significantly lower than that of the SSA-F1 and CY-F1 rats (Fig. 4C, left), whereas no differences were observed for *Gria4* (Fig. 4D, right). Additionally, we found that the levels of GABRG3 protein in the CSA-F1 rats were also significantly decreased compared to those of the SSA-F1 and CY-F1 rats (Fig. 4E, F). Taken together, these results indicate that the expression of *Gabrg3* in the VTA may be involved in regulating the cocaine-seeking motivation of male offspring rats.

### *Gabrg3* of VTA GABAergic neuron inhibits high incentive response to cocaine in male F1 rats

**Fig. 5:**
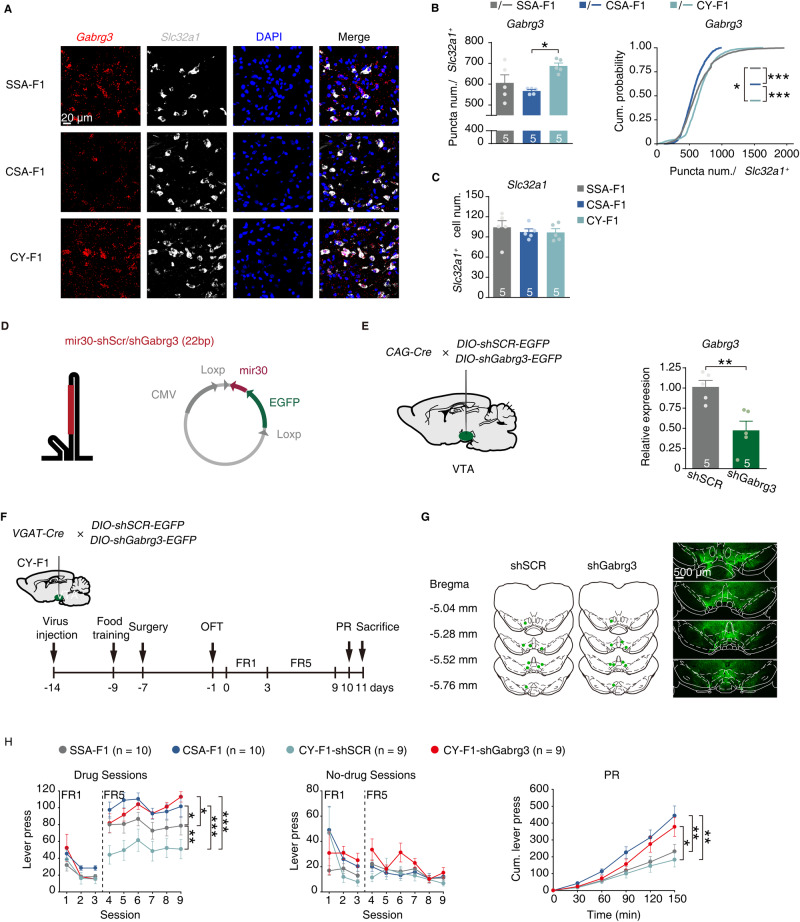
*Gabrg3* in VTA GABAergic neurons is involved in cocaine-seeking behavior. **A**–**C** Representative smFISH images (**A**), quantification of *Slc32a1*^+^ neurons (**B**), bar graph quantification (**C**, left), and cumulative frequency distribution (**C**, right) of *Gabrg3* mRNA levels in the VTA of CSA-F1, CY-F1, and SSA-F1 rats in *Slc32a1*^+^ neurons. SSA-F1, *n* = 5; CSA-F1, *n* = 5; CY-F1, *n* = 5. **D** Design of plasmid for knockdown of *Gabrg3* based on a miR-30 backbone in a *Cre*-dependent manner. **E** Schematic injection program (left) and qPCR assessment (right) for knockdown efficiency of GABRG3 in vivo. In vivo transfection was performed by injecting DOTAP with *pCAG-Cre* and *AAV-DIO-shSCR-EGFP* (shSCR) or *AAV-DIO-shGabrg3-EGFP* (shGabrg3) in VTA. shSCR, *n* = 5; shGabrg3, *n* = 5. **F** Schematic of virus injection and cocaine self-administration training. **G** Representative image of the expression of EGFP after virus injection in VTA. **H** Performance of cocaine self-administration in male F1 rats after knockdown of *Gabrg3*. Left, lever press in drug sessions under the FR program. Middle, lever press in no-drug sessions under the FR program. Right, cumulative lever press under the PR program. SSA-F1, *n* = 10; CSA-F1, *n* = 10; CY-F1-shSCR, *n* = 9; CY-F1-shGabrg3, *n* = 9. Results are shown as mean ± s.e.m., ***P < 0.001, **P < 0.01, *P < 0.05.

*Gabrg3*, which encodes the γ3 subunit of GABA_A_ receptors, has been associated with an increased risk of developing alcoholism [40, 41], autism, and other psychiatric disorders [42–45]. Previous reports have indicated that GABAergic input tends to activate GABA_A_ receptors in VTA GABAergic neurons, while it activates GABAB receptors in VTA dopaminergic neurons [46]. To determine whether changes in *Gabrg3* expression in the VTA of male F1 rats occur in GABAergic neurons, we used smFISH to quantify *Gabrg3* within *Slc32a1*^+^ cells (Fig. 5A–C). Indeed, while GABRG3 is widely distributed within the VTA, it aggregates in GABAergic neurons (Fig. 5A). And the expression level of *Gabrg3* in VTA GABAergic neurons of CSA-F1 rats was significantly lower than that of CY-F1 rats (Fig. 5B, C). In addition, no differences in the number of GABAergic neurons were observed between CSA-F1 and CY-F1 rats (Fig. 5C).

To evaluate the impact of *Gabrg3* in VTA GABAergic neurons on cocaine-seeking motivation in male F1 rats, we designed mir-30 based shRNA to knockdown the expression level of *Gabrg3* (shGabrg3) (Fig. 5D). We observed that shGabrg3 could decrease the expression level of *Gabrg3* by almost 50% in the VTA, compared to the scramble (Fig. 5E). Next, we injected the *AAV2/9-VGAT1-Cre-mCherry* with *AAV2/9-DIO-shGabrg3-EGFP*(CY-F1-shGabrg3) or *AAV2/9-DIO-shSCR-EGFP* (CY-F1-shSCR) into VTA of CY-F1 rats, followed by food training, open field test, and cocaine self-administration (Fig. 5F–H and Supplementary Fig. S5A, B). We observed no difference in food training and open field test between the CY-F1-shGabrg3 rats and CY-F1-shSCR rats, indicating that reducing the expression level of *Gabrg3* would not affect associative learning and anxiety level (Supplementary Fig. S5A, B). However, in cocaine self-administration training, compared to the CY-F1-shSCR rats, CY-F1-shGabrg3 rats displayed significantly higher lever presses in drug sessions of the FR5 program and higher cumulative lever presses in the PR test, achieving comparable performance with CSA-F1 rats (Fig. 5H). Therefore, the down-regulation of *Gabrg3* in VTA GABAergic neurons critically regulates cocaine-seeking motivation in CSA-F1 male offspring.

**Fig. 6:**
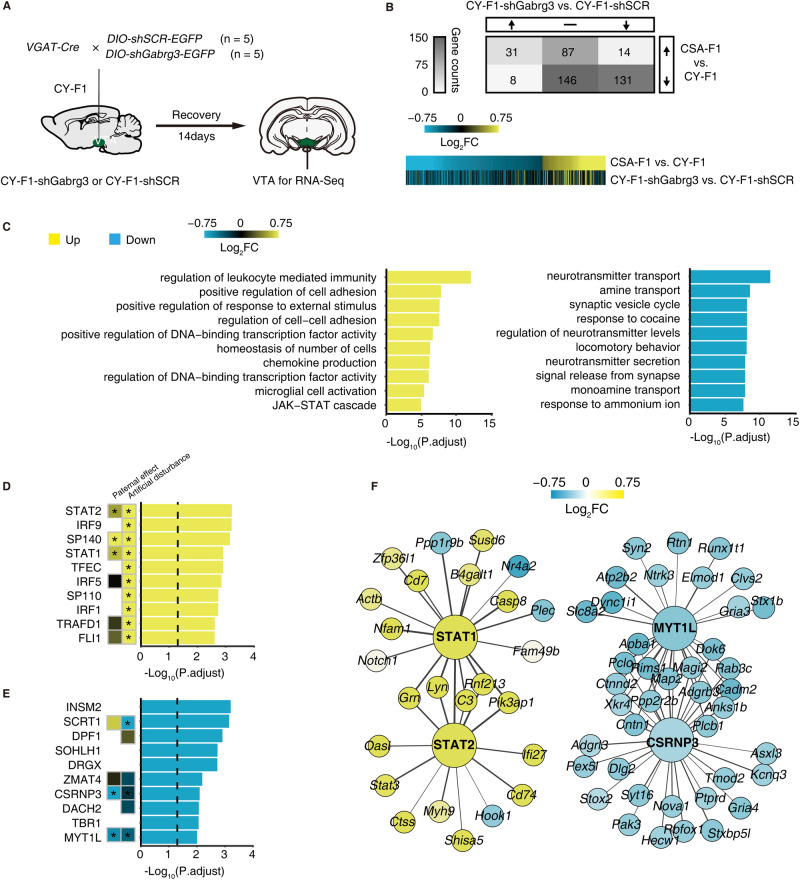
Knockdown of *Gabrg3* in VTA GABAergic neurons of CY-F1 reshapes the transcription network resembling CSA-F1. **A** Diagram of virus injection and RNA sequencing. **B** Tabular summary and heatmap showing DEGs between CSA-F1 vs. CY-F1 rats (top lane) and CY-F1-shGabrg3 vs. CY-F1-shSCR rats. **C**, **D** Top enriched GO analysis of upregulated and downregulated DEGs between CY-F1-shGabrg3 vs CY-F1-shSCR rats. **D**, **E** Top predicted upstream TFs of upregulated and downregulated DEGs between CY-F1-shGabrg3 vs CY-F1-shSCR rats. The TFs of CSA-F1 vs CY-F1 rats were also shown in the heat map and significantly changed TFs were marked. *P < 0.05. **F** Illustration of the gene network identified within DEGs of the CY-F1-shGabrg3 vs CY-F1-shSCR rats. Nodes were selected if they met the following criteria: 1. significantly changed in CSA-F1 vs. CY-F1 rats and CY-F1-shGabrg3 vs. CY-F1-shSCR rats; 2. predicted as downstream of TFs; 3. the connection with TFs in the network is of high importance (weight >0.35).

To investigate the regulatory mechanism underlying the increase in cocaine-seeking motivation observed in CY-F1 rats following the knockdown of *Gabrg3* in VTA GABAergic neurons, we performed transcriptome sequencing of VTA tissue from CY-F1 rats injected with the same viruses used in Fig. 5F (Fig. 6A). Consistent direction of changes in genes between CSA-F1 vs. CY-F1 and CY-F1-shGabrg3 vs. CY-F1-shSCR were observed, with 24.6% of the genes co-upregulated and 45.9% co-downregulated (Fig. 6B). Functional analysis of the regulated genes in the CY-F1-shGabrg3 revealed that, the knockdown of *Gabrg3* in VTA GABAergic neurons upregulated cell adhesion-related genes and downregulated genes related to synaptic neurotransmitter transmission (Fig. 6C). Furthermore, transcription factors enrichment was also similar between CSA-F1 vs. CY-F1 rats and CY-F1-shGabrg3 vs. CY-F1-SCR rats (Fig. 6D, E). Simultaneous transcription factor induced widespread downstream gene expression changes verified in gene expression networks (Fig. 6F). In summary, our findings suggest that the knockdown of *Gabrg3* in VTA GABAergic neurons in CY-F1 can remodel the transcriptional network of the VTA, inducing similar transcriptional changes observed in CSA-F1 rats, primarily affecting biological processes related to synapse organization and neurotransmitter transmission, and ultimately enhancing cocaine-seeking motivation in male F1 rats.

## Discussion

Research has shown a strong association between paternal drug addiction and offspring vulnerability to addiction in animal models [12–15]. However, many studies have used passive drug infusion methods, such as intraperitoneal injection, to investigate the effects of drug addiction on offspring. This is different from the active drug acquisition behavior observed in clinical practice among addicts [20, 47]. By establishing yoked-administration paradigms, our laboratory was able to better distinguish the effects of the drug itself from drug-seeking motivation. Consistent with the findings of Vassoler et al., we discovered that the offspring of rats that underwent cocaine self-administration exhibited a greater vulnerability to cocaine addiction, while the offspring of rats that underwent yoked-administration exhibited a decreased susceptibility to cocaine addiction [48].

In addition to vulnerability to addiction, paternal drug addiction can also impact cognitive and emotion-related behaviors in offspring. For instance, studies have shown that the offspring of male CD1 mice exposed to cocaine consumption exhibited impaired attention and spatial working memory [49], while the offspring of male LE rats exposed to subcutaneous infusion of cocaine demonstrated increased locomotor activity during adolescence [50]. However, in our study, we did not observe significant differences in male offspring of cocaine addicts during the dose-response test, behavioral sensitization, open field test, elevated plus maze, Y maze, new object recognition, and other behavioral tests. This may be due to differences in the genetic background among the experimental animals, as different rat strains may have varying innate behavioral responses to cocaine. Additionally, the differences in drug infusion patterns may result in differences in blood drug concentration during behavioral tests, which could also contribute to the lack of observed effects [51]. Cocaine induces sperm to undergo epigenetic reprogramming such as histone modification [52] and DNA methylation [48, 53]. As mentioned previously, during actual drug use the germ cells of drug addicts are challenged by mental stress in addition to drug stimulation, and this mental stress is also capable of inducing epigenomic reorganization in the germ cells [54]. Whereas the epigenome of germ cells under the combined effect of pharmacological and psychoactive factors may undergo a complex in-integration process [55], this process will probably not conform to a linear pattern. The specific manifestation of this is the difference in sperm epigenetic levels between addicted and non-addicted drug users [56]. This difference is likely to be reflected in gene expression levels in the brain regions of the offspring [53], triggering opposed drug-seeking phenotypic changes (Fig. 1).

Previous studies on the nervous system of offspring from drug abuse have primarily focused on non-specific brain regions and mechanisms. For example, chronic cocaine self-administration training in SD rats leads to decreased synaptic plasticity in the hippocampus of the offspring [22] and increased expression of adrenocorticotropic factor receptor 2 [57]. In addition, chronic alcohol vapor inhalation training in B6 mice results in increased expression of brain-derived neurotrophic factor in the VTA of offspring [18, 58]. Previous studies have struggled to effectively explain and eliminate the effects of paternal addiction on the drug-seeking motivation of offspring rats. Meanwhile, motivation is often believed to be closely related to the functional regulation of the VTA [36, 59]. To identify the key neurobiological mechanisms regulating cocaine-seeking motivation in offspring, we conducted transcriptome sequencing of the VTA in male F1 rats. We identified four gene modules related to the cocaine-seeking behavior of offspring rats, which are mainly involved in biological processes related to synaptic connection and extracellular matrix and are regulated by different hub genes and TFs. The strengthening of synaptic connections depends on various factors, including synaptic membrane receptors, astrocyte transmitter transport, and extracellular matrix [60], which is also consistent with our findings.

Using WGCNA methods, we identified *Gabrg3* as one of the hub genes, which has a strong association with Autism Spectrum Disorder [41] and alcohol dependence [61]. Moreover, *Gabrg3* is located in 15q11-q13, an intriguing locus linked to multiple psychiatric disorders, and is speculated to be an imprinted gene with maternal silencing and paternal expression [62, 63]. We confirmed the knockdown of *Gabrg3* in VTA GABAergic neurons of CSA-F1 rats through smFISH, resulting in enhanced cocaine-seeking motivation. Additionally, we found that the knockdown of *Gabrg3* in VTA GABAergic neurons of CY-F1 rats can remodel the transcriptional network in the VTA, causing it to exhibit “CSA-F1-like” changes. TFs such as MYT1L, CSRNP3, STAT1, and STAT2 showed the same differential changes between CSA-F1 and CY-F1 rats. Mammalian transcriptome studies have revealed that MYT1L is associated with GABA receptor-related genes [64]. MYT1L, known as the upstream trans-acting factor of membrane protein-related genes, can regulate many functions of membrane proteins [65]. These findings suggest that MYT1L and GABA_A_ receptors may play a vital role in regulating cocaine-seeking motivation in offspring rats. The relationship between the regulatory networks of these upstream TFs and drug-seeking motivation needs further exploration. Moreover, GABAergic neurons in the VTA primarily regulate the surrounding dopaminergic neurons, thus affecting the release of dopamine in many downstream brain regions. The connection between the activity changes of these downstream brain regions and drug-seeking motivation also needs to be further explored.

### Supplementary information

Supplement information

Key Resources Table

Supplemental Table 1

Supplemental Table 2

Supplemental Table 3

Statistical analysis

## Data Availability

All data are available in the main text or the supplementary materials. Additional data related to this paper may be requested from the authors. Raw NGS data are deposited in the NCBI BioProject database under accession numbers PRJNA788009 and PRJNA947380.
