# Supplementary material for: Paternal cocaine-seeking motivation defines offspring’s vulnerability to addiction by down-regulating GABAergic GABRG3 in the ventral tegmental area
Source: Transl Psychiatry. 2024 Feb 22;14:107. doi: 10.1038/s41398-024-02835-w (PMC10884401; doi:10.1038/s41398-024-02835-w)
Supplement: Supplementary file 1 — Supplement information [file 41398_2024_2835_MOESM1_ESM.pdf]

Supplementary Information for

Paternal cocaine-seeking motivation defines offspring's vulnerability to addiction by down-regulating GABAergic GABRG3 in the ventral tegmental area

This PDF file includes:

Materials and Methods

Figs. S1 to S7

References (1–9)

Materials and methods

Animals and housing

All experiments were conducted using Sprague-Dawley rats and their male offspring. Male and female Naïve rats were purchased from the Shanghai Center of Experimental Animals, Chinese Academy of Science, and acclimated to the facility for at least 1 week before any procedures. The rats were housed in groups of 4-6 in cages at a temperature of 23°C and a room humidity of around 30%, with a 12-hour reverse dark/light cycle (on at 4:00 PM, off at 4:00 AM), and had access to food and water ad libitum. All animal treatments in this study strictly comply with the National Institutes of Health Guide for the Care and Use of Laboratory Animals and were approved by the Animal Care and Use Committee of Shanghai Medical College at Fudan University

Surgery

Under isoflurane anesthesia, rats were implanted with a chronic indwelling jugular catheter (ID = 0.31 mm, OD = 0.64 mm, Dow Corning) into the right jugular vein and connected to a self-made pedestal mounted on the back. The rats were allowed to recover for seven days before behavioral experiments, and their catheter was flushed daily with 0.1 ml of saline containing heparin (30 IU/ml) and gentamicin (0.5 mg/ml) to maintain patency.

General procedures of operant conditioning behavior

Food training Rats were food-restricted to maintain 85% of their ad libitum body

weight and were then trained to press the active lever to obtain a 45 mg food pellet (Bio-Serv, Flemington, NJ, USA) in operant chambers (MED Associates Inc.) to facilitate the association between the lever and the reward. Rats that successfully obtained 150 food pellets were then subjected to surgery.

Fixed-ratio program Each operant chamber was equipped with two levers, one yellow light, one blue light, and a white cue light. Rats were trained to press the lever under a daily 2.5-hour fixed-ratio (FR) program of reinforcement, which consisted of three 40-minute drug-available sessions (drug sessions) separated by two 15-minute drug-unavailable sessions (no-drug sessions). Drug sessions were signaled by the blue light, and the number of lever presses during these sessions was used to assess the amount of drug intake. No-drug sessions were signaled by the yellow light, and the number of lever presses during these sessions was used to assess the compulsivity of drug-seeking. In drug sessions, rats pressed the active lever to reach a preset number, at which point an intravenous injection of the drug was delivered, the blue light was turned off, and a white cue light was turned on accompanied by a tone cue for 20 seconds. Each infusion was followed by a 20-second time-out period, during which the two levers were retracted. Pressing the inactive lever resulted in no programmed consequences at any time. In no-drug sessions, rats pressing the active lever resulted in no drug injection or conditioned cue.

Progressive-ratio program Rats were tested on a progressive ratio (PR) program to assess their drug-seeking motivation, during which the lever-press requirements for

each injection or pellet delivery (i) followed the formula i^{th} injection = Int ($5e^{0.25i-5}$), and the session stopped when rats took more than one hour to satisfy the response requirements. The cumulative lever presses were recorded every 30 minutes and used to assess drug-seeking motivation for each rat.

Yoked-administration program After food training and surgery, rats were randomly assigned to either perform voluntary drug self-administration or to receive passive drug yoked-administration. During self-administration training, two rats were randomly paired and self-administrating rats were allowed to freely press the lever for the infusion of cocaine or saline. On the other hand, yoked-administered rats received drug infusion passively at the same dose, time, and rate, without a lever, stretched out, and any conditioned cue, except for the blue light.

Intravenous cocaine self-administration and breeding scheme in F0 generation

After undergoing food training and surgery to implant intravenous catheters, the rats underwent self-administration training for 30 days. Group designation was randomly generated using sample() in the R language to select the animal numbers used in the experiment. The rats in the self-administration group received intravenous injections of cocaine (Coc-SA) or saline (Sal-SA) by voluntarily pressing the active lever, while the rats in the yoked-administration group were paired with the rats in the self-administration group to receive intravenous injections of cocaine (Coc-Yoke). Cocaine was administered at a dose of 500 $\mu g \cdot kg \cdot inf^{-1}$ for 4 seconds. The FR1 program was used for the first 5 days, and the Coc-SA rats could obtain an

intravenous injection of cocaine by pressing the active lever once; The FR5 program was used for the remaining 25 days, and the Coc-SA rats could obtain an intravenous injection of cocaine by pressing the active lever five times. The number of lever presses was used to assess the level of drug intake and the compulsivity of drug-seeking in rats. Two PR tests were performed on the 16th and 32nd days of training, and the rats' drug-seeking motivation was assessed by the cumulative number of lever presses (Fig. S1A).

The calculation formula of the behavior score (X) of each rat was $X = \frac{(Xi - \bar{X})}{s.d.}$. Xi represented the value of the behavior performance of each rat, $\bar{X}$ was the mean value of the behavioral performance of all the rats, and s.d. was the standard deviation of the value of the behavioral performance of all the rats. The drug-seeking compulsivity score (compulsivity score) was normalized by the mean number of lever presses in the no-drug sessions of the FR training, the drug-seeking motivation score (motivation score) was normalized by the cumulative number of lever presses in the PR test, and the drug intake score (intake score) was normalized by the mean number of lever presses during the drug sessions of FR training. The compulsivity score and the motivation score were added to obtain an addiction-like behavior score (addiction-like score). The F0 rats of the cocaine self-administration group (CSA-F0) were selected from the rats in the top 20% of the addiction-like score. The F0 rats in the yoked-administration group (CY-F0) were selected from the rats paired with CSA-F0 rats, while the F0 rats in the saline self-administration group (SSA-F0) were

randomly selected from the saline self-administration group. 24 hours after the last self-administration training, each selected F0 rat was mated with two naïve female rats to generate F1 rats. In all experiments, 2-3 male rats from each litter were randomly selected for the experiments. After behavioral tests, rats in poor postoperative conditions, such as catheter blockage and diarrhea, were excluded from the experiments.

Intravenous cocaine self-administration tests of male F1 rats

The F1 rats used for testing in this experiment were randomly selected, and we used sample() in the R language to select the animal numbers used in the experiment. The double-blind method was used in this experiment during the behavioral analysis of F1 rats. Animal experiments and data analysis were performed by independent experimenters, and the groups of animals were revealed after completion of the analysis. After undergoing food training and surgery for the implantation of an intravenous catheter, rats were initially trained to press the lever for cocaine using the FR1 program for three sessions. They were then trained using the FR5 program for six sessions. Finally, the rats were subjected to a PR program test.

For dose-response curve tests, the rats were trained to press the lever to receive descending doses of cocaine (750, 500, 300, 75, 50, 30, 22.5, 0 $\mu g \cdot kg \cdot inf^{-1}$, administered intravenously) using the FR5 program. Each dose was maintained for at least three days and until the lever presses remained stable over two days with less

than a 10% variation. The mean lever presses from the last two days of each dose were recorded.

Open field test

The open field test was primarily used to assess both locomotor activity and anxiety-like behavior in animals. Normally, animals tend to explore the central area of the field, while animals exhibiting anxiety-like behaviors tend to hide in the surrounding corners. Rats were placed in a rectangular behavior chamber (60 cm × 60 cm × 60 cm, L × W × H), and the light intensity in the center of the chamber was set to 60 lux. One week before the experiment, the rats were allowed to adapt to grasping or intraperitoneal injection. During the experiment, the rats were either intraperitoneally injected with cocaine or saline according to the experimental requirements or directly placed into the behavior chamber. Their movements were recorded for 15 minutes, and a camera located above the chamber recorded the position of each rat during the experiment. Finally, the total distance traveled, entries, and time spent in the central area (30 cm × 30 cm, L × W) by the rats were analyzed using Ethovision XT software. After the training of each rat was completed, the behavior chamber was wiped with 75% ethanol to remove any odor.

Behavior sensitization test

Repeatedly injecting the same dose of psychostimulant drugs can make drug-induced changes in locomotor activity more pronounced, a phenomenon known as behavioral sensitization. This method is commonly used to evaluate the response to

the positive reinforcement effect of psychostimulant drugs.

In this experiment, rats were placed in a rectangular behavior chamber (60 cm × 60 cm × 60 cm, L × W × H), and the light intensity in the center of the chamber was set to 60 lux. One week before the experiment, the rats were allowed to adapt to grasping and intraperitoneal injection. During the experiment, the rats were intraperitoneally injected with cocaine or saline, and their movements were recorded for 15 minutes. Saline was injected intraperitoneally for the first two days, followed by repeated intraperitoneal injections of cocaine (5 or 10 mg/kg) for five consecutive days. After a 10-day withdrawal period, the rats were given cocaine (5 or 10 mg/kg) to measure the drug-induced alteration in locomotor activity. A camera located above the chamber recorded the movements of the rats during each experiment. Finally, the total distance traveled by the rats was analyzed using Ethovision XT software, and the sensitization index was calculated to confirm the occurrence of behavioral sensitization. After the training of each rat was completed, the behavior chamber was wiped with 75% ethanol to remove any odor.

Elevated plus maze test

The elevated plus-maze test is primarily used to evaluate anxiety-like behavior in animals. Typically, normal animals will explore the open arm, while animals with anxiety-like behavior will prefer to stay in the closed arm. The experimental platform is 50 cm above the ground and includes two closed arms (30 cm × 10 cm × 30 cm, L × W × H), two open arms (30 cm × 10 cm, L × W), and a middle area (10 cm × 10 cm,

L=× W). At the beginning of the experiment, the rat is placed in the middle area of the maze, facing the open arm, and allowed to freely explore for 5 minutes. The camera above the maze records the rat's traveled trajectory. Finally, the total travel distance, entries, and time spent in the open arm are analyzed using Ethovision XT software. After the training of each rat, the behavioral chamber is wiped with 75% ethanol to remove any odors.

Novel object recognition test

Novel object recognition experiments are typically used to evaluate animals' learning and memory abilities. Normal animals tend to explore new objects for a long time due to their exploratory nature, while animals with impaired learning and memory abilities cannot effectively distinguish between old and new objects. Rats were placed in a rectangular behavior chamber (measuring 60 cm × 60 cm × 60 cm, L × W × H) three days before training and were acclimated for 10 minutes each day. At the start of the experiment, two objects made of the same material and shape were placed symmetrically in opposite corners of the chamber (5 cm from the wall). Rats were allowed to explore the objects freely for 10 minutes, after which they were returned to their cages. After 24 hours, one of the familiar objects was replaced with a new object made of the same material but a different shape, and the rats were again allowed to explore the objects freely for 10 minutes. A camera positioned above the behavior chamber recorded the rats' positions. The time and number of times that the rats explored each object were analyzed using Ethovision XT software, and the

discrimination index (DI) of each rat was calculated. "Exploring" was defined as rats placing their noses within 2 cm of an object or touching an object with their nose or front paws. The discrimination index was calculated using the following formula: DI = (Time novel − Time familiar)/ (Time novel + Time familiar). After completing the training for each rat, the behavioral chamber was wiped down with 75% ethanol to remove any odors.

Y-maze

The Y-maze experiment is primarily used to evaluate experimental animals' spatial memory abilities. The apparatus is constructed from PMMA and consists of three arms (measuring 30 cm × 10 cm × 30 cm, L × W × H) set at a 120-degree angle to each other. Normal animals tend to explore the novel arm for a longer period due to their exploratory nature, whereas animals with impaired spatial memory can not effectively distinguish between the three arms. For each rat, the three arms are randomly designated as the familiar arm, start arm, and novel arm. Rats are placed in the testing room for at least 1 hour before the start of the experiment. During training, the rat is placed in the start arm and allowed to explore the start arm and familiar arm for 10 minutes while the novel arm is blocked off. One hour later, all arms are made available, and the rat is allowed to explore the maze for 5 minutes. A camera positioned above the maze records the rats' trajectory and positions. The time spent in and the entries into the novel arm are analyzed using EthoVision XT software to calculate the error rate of selecting the novel arm. The error rate is calculated as the

entries into the novel arm divided by the entries into all arms, multiplied by 100%. After completing the training for each rat, the apparatus is wiped down with 75% ethanol to remove any odors.

RNA extraction

The rats were anesthetized with isoflurane and euthanized by transcardial perfusion with 1× phosphate-buffered saline, followed by decapitation. The brains were rapidly extracted and placed ventral surface up onto a chilled brain matrix on ice. The rostral and caudal borders of the VTA were visually located according to the brain atlas, and a ~1 mm-thick coronal section was obtained using a chilled razor blade. The VTA was then microdissected at its dorsolateral borders according to the boundary characteristic and homogenized in RNA isolation Total RNA Extraction Reagent (Vazyme), followed by the addition of chloroform and centrifugation. After the supernatant was separated, isopropanol and linear acrylamide were added, mixed well, and then centrifuged after being placed at -80°C for 2 hours. The RNA pellet was resuspended with 75% ethanol and the ethanol was discarded after centrifugation. The pellet was air-dried for ethanol and then dissolved with RNase-free water before being placed at -20°C for a short time for subsequent experiments.

Library preparation and sequencing

The concentration and quality of purified total RNA were measured using the Qubit 3.0 and Agilent 2100 Bioanalyzer. Total RNA libraries were created using the Ribo-off rRNA Depletion Kit (Human/Mouse/Rat) (Vazyme) and Total RNA-seq

(H/M/R) Library Prep Kit for Illumina (Vazyme), following the manufacturer's instructions. In brief, 300 ng total RNA was mixed with an rRNA probe to create an rRNA-probe hybrid, which was then depleted by RNase H. The probe was then removed using DNase I digestion. The rRNA-depleted RNA was fragmented and the general library was prepared by adding dNTP in cDNA synthesis and ligation, along with RNA adapters. Size selection was performed to enrich the insertion size range from 200 bp to 450 bp. Sequencing was carried out on the Hiseq 3000 using a dual barcoding index for multiplexing samples, and the sequencing depth was an average of 50 million reads.

Bioinformatics analysis

The raw sequencing reads were first processed using Trimmomatic (V0.32) [1] to remove adapters and filter out low-quality reads (Phred score > 28, > 50 bp, no unusual sequence repeats). The resulting clipped reads were then aligned to the rat genome (Ensembl Rnor_6.0) using Hisat 2 (version 2.0.2) [2], and gene annotation and read counting were performed using featureCounts [3]. DEGs were performed using DESeq2 (version 1.26.0) [4], with DEGs defined as those with a *P*-value ≤ 0.05 and mean counts ≥ 80. Gene co-expression network analysis was carried out using WGCNA(version 1.70-3) [5], with highly co-regulated genes identified using average linkage-hierarchical clustering and a dynamic cut-tree algorithm. Hub genes were defined as those with k_{total} > 20 and weight > 0.4 produced by WGCNA intramodular connectivity. Network graphs were generated using Cytoscape (version 3.7.2). TFs

enrichment analysis was performed using ChEA3 [6], and gene ontology annotation and gene set enrichment analysis was carried out using the R package “clusterProfiler” [7]. Multiple gene modules gene ontology annotation was performed using ClueGO [8].

smFISH by RNAscope

The rats, aged 7-8 weeks, were perfused with phosphate-buffered saline and a 4% paraformaldehyde solution. The brain was then removed and post-fixed in a 4% paraformaldehyde solution for 12 hours. The brain was dehydrated twice in 30% sucrose in phosphate-buffered saline. The smFISH was performed on frozen brain slices containing the VTA, which were 10 micrometers thick, following the RNAscope procedures (Advanced Cell Diagnostics).

Briefly, frozen slices containing the VTA were thaw-mounted onto SuperfrostTM Plus Microscope Slides (Thermofisher) and pretreated with protease digestion for 20 minutes at room temperature. Slices were then incubated with probes targeting "*Gabrg3* and *Gria4*" or "*Gabrg3* and *Slc32a1*" for 2 hours at 40°C with a labeled probe mixture per slide (*Gabrg3*, accession No: NM_024370, target region 121-1257; *Gria4*, accession No: NM_017263.6, target region 453-1831; *Slc32a1*, accession No: NM_031782.1, target region 288-1666). The nonspecifically hybridized probe was removed by washing the slices in washing buffer at room temperature, followed by Amplifier 1-FL for 30 minutes, Amplifier 2-FL for 30 minutes, and Amplifier 3-FL for 15 minutes at 40°C. Each amplifier was removed by washing with a washing buffer for 2 minutes at room temperature. At least four brain slices from each rat

underwent smFISH and were imaged.

Western blotting

Tissue (100 mg) was isolated in RIPA lysis buffer (1 ml, ThermoFisher) containing protease inhibitor cocktail (Sigma) and homogenized for protein extraction. The tissue homogenates were placed on ice for 30 minutes and then centrifuged at 30,000 g for 30 minutes at 4°C. The supernatant was collected for protein quantification using the Pierce BCA Protein Assay Kit (ThermoFisher).

For immunoblotting, 20 μg of protein was loaded per lane for gel electrophoresis onto a 10% SDS-polyacrylamide gel and transferred to polyvinylidene difluoride membranes (Beyotime). The membranes were blocked with 5% (w/v) milk in TBS containing 0.1% Tween-20 (TBST) and incubated overnight with the primary polyclonal antibody GABRG3 (1:1000; ThermoFisher). After washing with TBST, the membranes were incubated for 1 hour with secondary antibodies conjugated with the IRDye800 or IRDye700 (Maravai). Images were captured using Odyssey CLx (LI-COR Biosciences) and quantified with Image Studio V3.1.

Plasmid design and validation of the effectiveness

To achieve Cre-dependent gene downregulation, a synthetic miR-30 based framework that downregulates *Gabrg3* (TGGTCCTGTGTCATCCATAAAC) or a scramble sequence (TCTTGAGTGTTCCAAACCATCG) into *pAKD-CMV-bGlobin-Flex-EGFP-MIR30shRNA* plasmid using *EcoRI/XhoI* restriction sites[9].

The expression efficacy of the plasmid that downregulates *Gabrg3* or the scramble sequence was validated using the DOTAP transfection system (Roche). Briefly, the plasmid concentration was diluted to 1 μg/ml, and the *pCDNA3.0-CAG-Cre* plasmid and the plasmid that downregulates *Gabrg3* or the scramble sequence were mixed at a ratio of 1:1. The DOTAP transfection reagent and the plasmid mix were transfected at a ratio of 1:6 to the VTA, and real-time quantitative PCR analysis of *Gabrg3* expression was performed to verify plasmid expression and knock-down efficiency 120 hours after transfection. The follow-up virus packaging was completed by Shanghai Obio Biotechnology Co., Ltd.

Quantitative PCR analysis

After RNA extraction, a 6.5 μl reaction mixture containing 300 ng total RNA and random hexamer primers was heated at 65 °C for 2 minutes and immediately cooled down on the ice for 2 minutes. Then, 0.5 μl of 10 mM dNTPs, 0.5 μl of reverse transcriptase, 0.5 μl of RNase inhibitor, and 2 μl of 5× RT buffer were added. The reverse transcription program was carried out in a PCR machine as follows: 25 °C for 5 minutes, 50°C for 30 minutes, 85 °C for 2 minutes, and held at 4 °C.

Real-time quantitative PCR was performed as follows: the 20 μl reaction contained 2 μl of reverse transcription products, 200 nM of each primer, 10 μl of 2× SYBR Green PCR master mix, and water. The thermal cycler program was as follows: 95°C for 5 minutes, followed by 40 cycles at 95 °C for 10 seconds, and then 60 °C for 30 seconds. All reactions were run in triplicate.

Target Gene	Sequence	
Gabrg3	F-	AAACCAGAAGTGGGTCTTGG
	R-	GTTGAATCGAAGGCGACTG
Gapdh	F-	GCATCTTCTTGTGCAGTGCC
	R-	GATGGTGATGGGTTTCCCGT

Virus preparation and stereotaxic injection

AAV2/9-VGAT1-Cre-mCherry-WPRE-hGH-pA was purchased from BrainVTA (Wuhan) Technology Corp., Ltd. *AAV2/9-DIO-shGabrg3-EGFP* and *AAV2/9-DIO-shSCR-EGFP* were packaged by Shanghai Obio Biotechnology Co., Ltd.

The rats were anesthetized with isoflurane and placed in a stereotaxic instrument. Holes were drilled in the skull at the targeted coordinates, and all infusions were performed bilaterally using glass pipettes and a Drummond Nanoject III at a delivery rate of 4 μl·s^{-1}. *AAV2/9-VGAT1-Cre-mCherry-WPRE-hGH-pA* and *AAV2/9-DIO-shGabrg3-EGFP* or *AAV2/9-DIO- shSCR-EGFP* were mixed to equal particle numbers before injection. The intended stereotaxic coordinates for the VTA were AP: - 5.6 ~ -5.2 mm, ML: ± 0.8 mm, DV: - 7.5 ~ -7.3 mm. After completing the injection, the glass pipette was left in the brain for an additional 10 min to ensure sufficient diffusion. The virus-injected animals were allowed to recover for at least 7 days. After behavioral tests, brain slides that included the VTA were collected to

verify the expression of EGFP. Rats with incorrect coordinates were excluded.

Statistical analysis.

All experimental data were analyzed using GraphPad Prism 9, Stata 16, and the R programming language for statistical analysis and graphing. To assess the assumption of homogeneity of variances, we conducted Levene's test for homogeneity of variances. Behavioral data on cocaine self-administration during FR and PR programs were analyzed using mixed linear models with repeated measurements (MMRM) and pairwise comparison post hoc tests. Multiple group comparisons were analyzed using one-way or two-way analysis of variance (ANOVA) and Bonferroni's multiple comparison post hoc test. Nonparametric tests were used when the data did not follow a normal distribution. Sample size were pre-estimated based on our previous experiences[10], and estimation was also conducted with an alpha value of 0.05 and a desired power of 0.80. A significance level of $P < 0.05$ was used. The data are presented as mean ± s.e.m.

Legends for tables and figures

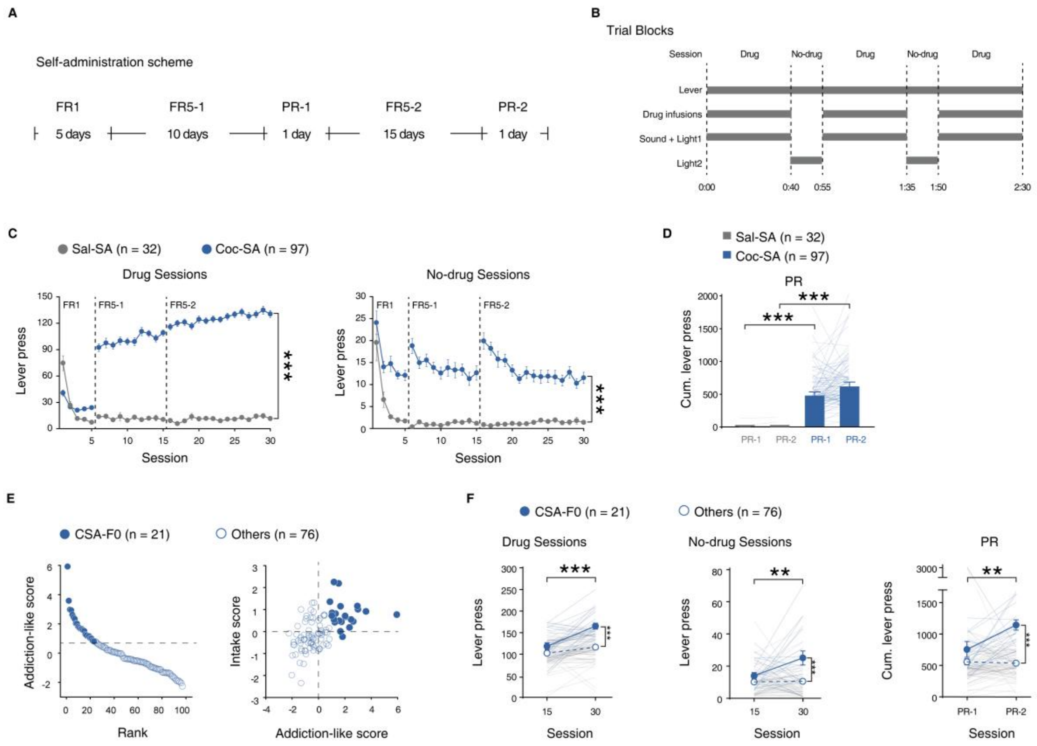

Figure S1. Performance of F0 rats in cocaine self-administration training.

(A) The 30 days training consisted of a 5-day FR1, 25-day FR5, and 2-day PR test.

(B) The within-session self-administration training protocol.

(C) Lever presses of Coc-SA and Sal-SA rats in drug sessions (left) and no-drug sessions (right) of the FR program. Sal-SA, n = 32; Coc-SA, n = 97.

(D) Cumulative lever presses under the PR program test on day 16 (PR-1) and day 31 (PR-2). Sal-SA, n = 32; Coc-SA, n = 97

(E) Scatter plot of addiction-related behavior scores of each Coc-SA rat. Left: the top 20% of the addiction-like scores from Coc-SA rats were selected as CSA-F0 (blue-filled circle). Right: These selected Coc-F0 rats also had higher intake scores during the cocaine self-administration training. CSA-F0, n = 21; Others, n = 76.

(F) Comparison of lever presses between CSA-F0 (blue filled) with others (blue hollow circles) in drug sessions (left), no-drug sessions (middle) of FR program on day 15 and day30, and cumulative lever presses on PR-1 and PR-2 test (right). CSA-F0, n = 21; Others, n = 76.

Results are shown as mean ± s.e.m, ***$P < 0.001$, **$P < 0.01$, *$P < 0.05$.

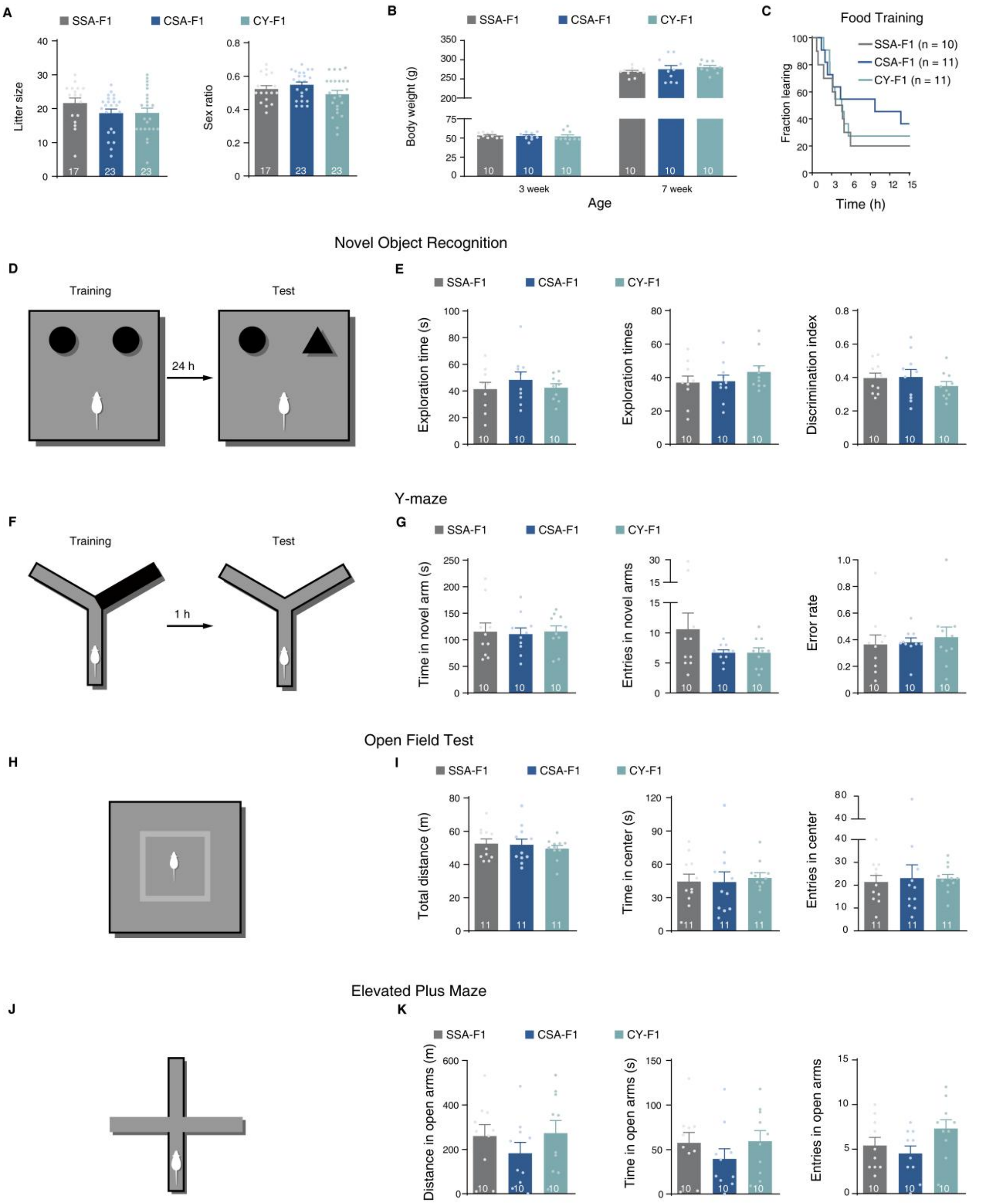

Fig S2. Comparable performance of cognitive and emotion-related behavior in male F1 rats.

(A) Litter size and gender ratio of male F1 rats. SSA-F1, n = 17; CSA-F1, n = 23; CY-F1, n = 23.

(B) Body weight of male F1 rats at 3 and 7 weeks of age. SSA-F1, n = 10; CSA-F1, n = 10; CY-F1, n = 10.

(C) Learning curve of male F1 rats in operant conditioning tasks to obtain food pellets. SSA-F1, n = 10; CSA-F1, n = 11; CY-F1, n = 11.

(D, E) Novel object recognition test of male F1 rats. (D) Experimental schematic diagram. (E) Performance in the novel object recognition test of CSA-F1, CY-F1, and

SSA-F1 rats. Left, time spent exploring the novel object. Middle, number of times the novel object was explored. Right, discrimination index for novel objects. SSA-F1, n = 10; CSA-F1, n = 10; CY-F1, n = 10.

(F, G) Y maze test of male F1 rats. (F) Experimental schematic diagram. (G) Performance in the Y maze of CSA-F1, CY-F1, and SSA-F1 rats. Left, time spent exploring the novel arm. Middle, entries into the open arms. Right, the error rate of entering the novel arm. SSA-F1, n = 10; CSA-F1, n = 10; CY-F1, n = 10.

(H, I) Open field test of male F1 rats. (H) Experimental schematic diagram. (I) Performance in the open field test of CSA-F1, CY-F1, and SSA-F1 rats. Left, total distance traveled. Middle, time spent in the central area. Right, entries into the central area. SSA-F1, n = 10; CSA-F1, n = 10; CY-F1, n = 10.

(J, K) Elevated plus maze of male F1 generation rats. (J) Experimental schematic diagram. (K) Performance in the elevated plus maze of CSA-F1, CY-F1, and SSA-F1 group rats. Left, total distance traveled in the open arms. Middle, time spent in the open arms. Right, entries into the open arms. SSA-F1, n = 10; CSA-F1, n = 10; CY-F1, n = 10.

Results are shown as mean ± s.e.m, ***$P < 0.001$, **$P < 0.01$, *$P < 0.05$.

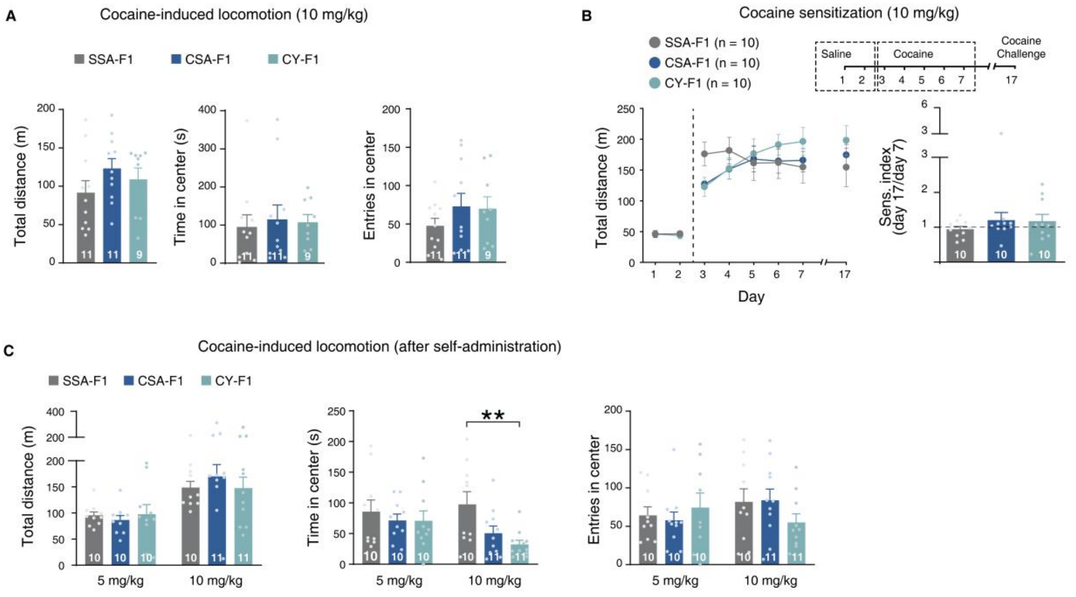

Figure S3. Comparable performance of response to cocaine under different doses in male F1 rats.

(A) Cocaine-induced locomotion activity of naïve male F1 rats under 10 mg/kg doses. Left, total distance traveled. Middle, time spend in the center area, Right, entries to the center area. SSA-F1, n = 11; CSA-F1, n = 11; CY-F1, n = 9.

(B) Behavioral sensitization of male F1 rats under 10 mg/kg doses. Left, total distance traveled; Right, sensitization index during the training session with 10 mg/kg cocaine injections (IP). SSA-F1, n = 10; CSA-F1, n = 10; CY-F1, n = 10.

(C) Cocaine-induced locomotion activity of male F1 rats after cocaine self-administration training under 5 mg/kg and 10 mg/kg doses. Left, total distance traveled. Middle, time spend in the center area, Right, entries to the center area. 5 mg/kg: SSA-F1, n = 10; CSA-F1, n = 10; CY-F1, n = 10. 10 mg/kg: SSA-F1, n = 10; CSA-F1, n = 11; CY-F1, n = 11.

Results are shown as mean ± s.e.m, $^{***}P < 0.001$, $^{**}P < 0.01$, $^{*}P < 0.05$.

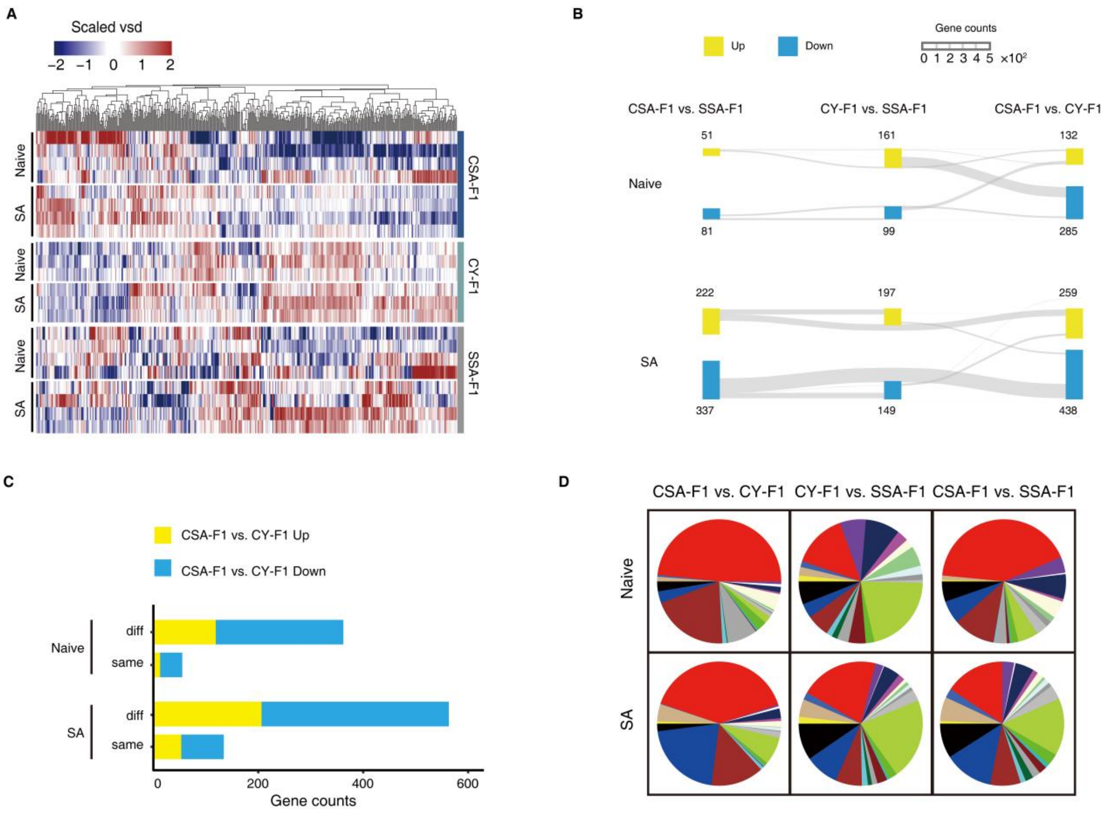

Figure S4. Analysis of the relationship between changes in gene expression and gene co-expression networks.

(A). The heatmap shows the variation-stabilizing transformed (VST) expression pattern of male F1 rats under Naive and SA states.

(B). Counts of DEGs across male F1 rats. The same genes are connected with gray ribbons.

(C). Based on the direction of expression change in SSA-F1 rats, we defined genes that $Log_2FC_{CSA\text{-}F1\ vs.\ SSA\text{-}F1} \times Log_2FC_{CY\text{-}F1\ vs.\ SSA\text{-}F1} > 0$ as the same direction changed genes, while genes that didn't meet this criterion were defined as different direction changed genes. The bar plot shows that the majority of DEGs belong to different direction changed genes.

(D). Pie charts showing the module constitution of DEGs.

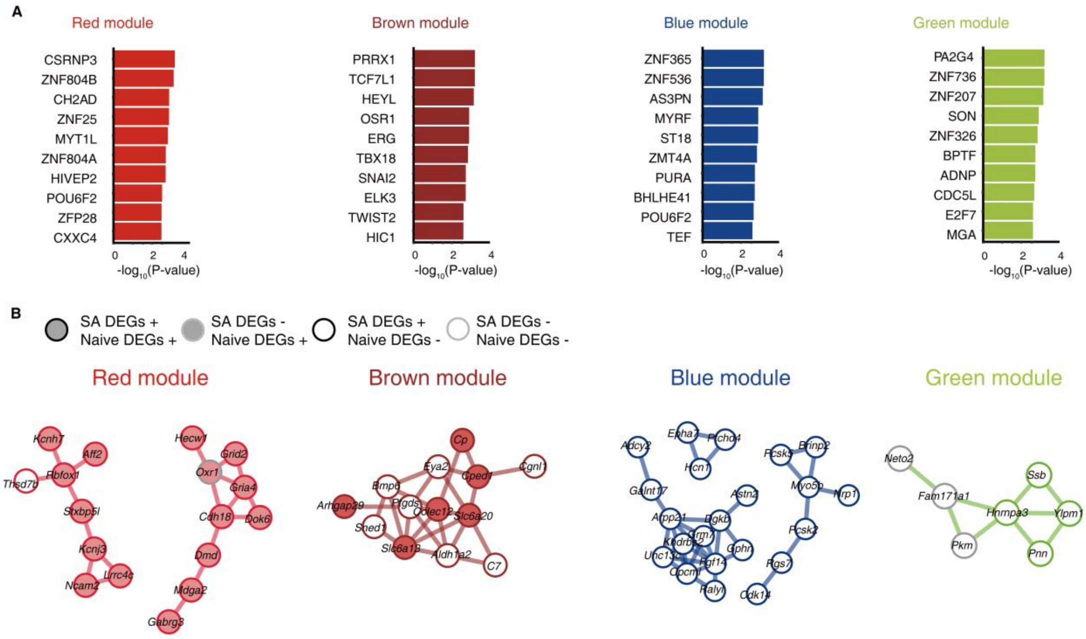

Figure S5. Analysis of the relationship between changes in gene expression and gene co-expression networks.

(A). Top 10 predicted TFs of red, brown, blue and green modules, arranged by significance.

(B). Hub genes of red, brown, blue and green modules based on intramodule connectivity. Circles with borders, DEGs underSA state; filled circle, DEGs under naïve. state.

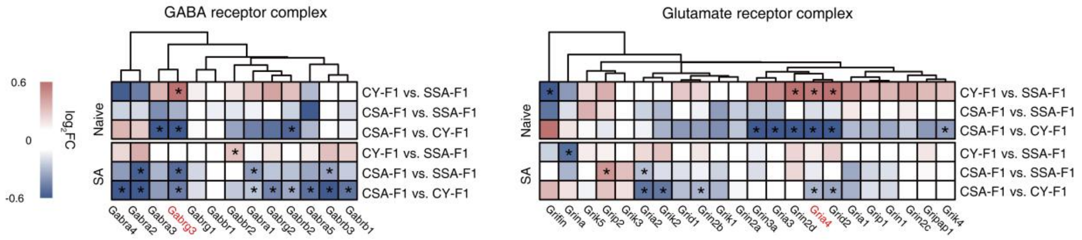

Figure S6. Transcriptional changes of GABA and Glutamate receptor complex. The heatmap shows the between-group differences in expression of GABA and glutamate receptor complex, red = up-regulation, blue = down-regulation, $*P < 0.05$.

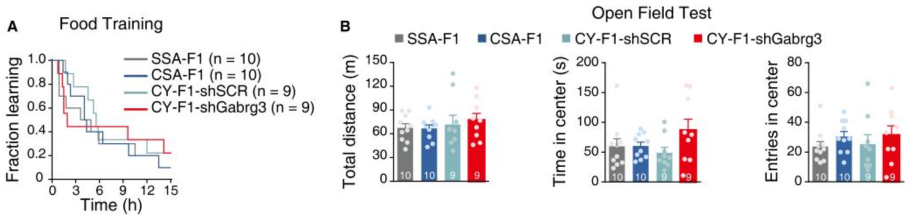

Figure S7. Behavior performance in operant behavior learning and open filed tests of male F1 rats under VTA GABAergic *Gabrg3* knock-down.

(A, B) Behavior performance of CSA-F1, SSA-F1, and CY-F1 group rats injected with scramble (CY-F1-shSCR) or shGabrg3 (CY-F1-shGabrg3). SSA-F1, n = 10; CSA-F1, n = 10; CY-F1-shSCR, n = 9; CY-F1-shGabrg3, n = 9.

(A) Learning curve of CSA-F1, SSA-F1, CY-F1-shSCR, and CY-F1-shGabrg3 rats in operant conditioning tasks to obtain food pellets.

(B) Open field test of CSA-F1, SSA-F1, CY-F1-shSCR, and CY-F1- shGabrg3 rats. Left, total distance traveled. Middle, time spend in the center area, Right, entries to the center area.

References

1 Bolger, A. M., Lohse, M. & Usadel, B. Trimmomatic: a flexible trimmer for Illumina sequence data. *Bioinformatics* **30**, 2114-2120 (2014). https://doi.org:10.1093/bioinformatics/btu170

2 Kim, D., Paggi, J. M., Park, C., Bennett, C. & Salzberg, S. L. Graph-based genome alignment and genotyping with HISAT2 and HISAT-genotype. *Nat Biotechnol* **37**, 907-915 (2019). https://doi.org:10.1038/s41587-019-0201-4

3 Liao, Y., Smyth, G. K. & Shi, W. featureCounts: an efficient general purpose program for assigning sequence reads to genomic features. *Bioinformatics* **30**, 923-930 (2014). https://doi.org:10.1093/bioinformatics/btt656

4 Love, M. I., Huber, W. & Anders, S. Moderated estimation of fold change and dispersion for RNA-seq data with DESeq2. *Genome Biol* **15**, 550 (2014). https://doi.org:10.1186/s13059-014-0550-8

5 Zhang, B. & Horvath, S. A general framework for weighted gene co-expression network analysis. *Stat Appl Genet Mol Biol* **4**, Article17 (2005). https://doi.org:10.2202/1544-6115.1128

6 Keenan, A. B. *et al.* ChEA3: transcription factor enrichment analysis by orthogonal omics integration. *Nucleic Acids Research* **47**, W212-W224 (2019). https://doi.org:10.1093/nar/gkz446

7 Yu, G., Wang, L. G., Han, Y. & He, Q. Y. clusterProfiler: an R package for comparing biological themes among gene clusters. *OMICS* **16**, 284-287 (2012). https://doi.org:10.1089/omi.2011.0118

8 Bindea, G. *et al.* ClueGO: a Cytoscape plug-in to decipher functionally grouped gene ontology and pathway annotation networks. *Bioinformatics* **25**, 1091-1093 (2009). https://doi.org:10.1093/bioinformatics/btp101

9 Chang, K., Marran, K., Valentine, A. & Hannon, G. J. Creating an miR30-based shRNA vector. *Cold Spring Harb Protoc* **2013**, 631-635 (2013). https://doi.org:10.1101/pdb.prot075853

10 Le, Q. *et al.* Drug-seeking motivation level in male rats determines offspring susceptibility or resistance to cocaine-seeking behaviour. *Nature communications* **8**, 1-13 (2017).
